# AI-augmented physics-based docking for antibody-antigen complex prediction

**DOI:** 10.1093/bioinformatics/btaf129

**Published:** 2025-03-26

**Authors:** Francis Gaudreault, Traian Sulea, Christopher R Corbeil

**Affiliations:** Human Health Therapeutics Research Centre, National Research Council Canada, Montreal, Quebec H4P 2R2, Canada; Human Health Therapeutics Research Centre, National Research Council Canada, Montreal, Quebec H4P 2R2, Canada; Institute of Parasitology, McGill University, Sainte-Anne-de-Bellevue, Quebec H9X 3V9, Canada; Human Health Therapeutics Research Centre, National Research Council Canada, Montreal, Quebec H4P 2R2, Canada; Department of Biochemistry, McGill University, Montreal, Quebec H3A 1A3, Canada

## Abstract

**Motivation:**

Predicting the structure of antibody-antigen complexes is a challenging task with significant implications for the design of better antibody therapeutics. However, the levels of success have remained dauntingly low, particularly when high standards for model quality are required, a necessity for efficient antibody design. Artificial intelligence (AI) has significantly impacted the landscape of structure prediction for antibodies, both alone and in complex with their antigens.

**Methods:**

We utilized AI-guided antibody modeling tools to generate ensembles displaying diversity in the complementarity-determining region (CDR) and integrated those into our previously published AlphaFold2-rescored docking pipeline, a strategy called AI-augmented physics-based docking. In this study, we also compare docking performance with AlphaFold and Boltz-1, the new state-of-the-art. We distinguish between two types of success tailored to specific downstream applications: (i) criteria sufficient for epitope mapping, where gross quality is adequate and can complement experimental techniques, and (ii) criteria for producing higher-quality models suitable for engineering purposes.

**Results:**

We highlight that the quality of the ensemble is crucial for docking performance, that including too many models can be detrimental, and that prioritization of models is essential for achieving good performance. In a scenario analogous to docking using a crystallized antigen, our results robustly demonstrate the advantages of AI-augmented docking over AlphaFold2, further accentuated when higher standards in quality are imposed. Docking also shows improvements over Boltz-1, but those are less pronounced. Docking performance is still noticeably lower than AlphaFold3 in both epitope mapping and antibody design use cases. We observe a strong dependence on CDR-H3 loop length for physics-based tools on their ability to successfully predict. This helps define an applicability range where physics-based docking can be competitive to the newer generation of AI tools.

**Availability and implementation:**

The AF2 rescoring scripts are available at github.com/gaudreaultfnrc/AF2-Rescoring.

## 1 Introduction

Predicting the structure of antibody-antigen complexes has tremendous value in biomedical research, with implications in understanding immune responses and designing therapeutics. While recent years have seen a growing reliance on sequence-based approaches for antibody research (Shuai *et al.* 2023, Hie *et al.* 2024), access to structural information remains crucial for applications requiring the spatial arrangements of protein atoms such as affinity maturation (Vivcharuk *et al.* 2017) and developability improvement (Chennamsetty *et al.* 2009, Zambrano *et al.* 2015, Agrawal *et al.* 2016). Accurate predictions of antibody-antigen complexes at atomic level remains a challenging task. Predicted structural models often come with uncertainties, emphasizing a persistent need for developing more accurate modeling tools. Recent advancements in deep learning, notably with the emergence of AlphaFold (Jumper *et al.* 2021, Evans *et al.* 2022), have renewed the optimism for improvements in protein structural prediction. Although AlphaFold has demonstrated an unprecedented performance in predicting generic protein–protein complexes, it has shown lower success rates against antibody-antigen complexes (Yin *et al.* 2022), primarily due to the absence of co-evolutionary constraints.

Alternative methods exist to predict structures of complexes such as traditional physics-based molecular docking tools (Chen *et al.* 2003, Dominguez *et al.* 2003, Hogues *et al.* 2018). In the realm of molecular docking, a prevalent limitation arises from the lack of appropriate simulations of protein backbone flexibility, thus constraining the accuracy and the applicability of docking (Huang 2014). The significance of this limitation is underscored in the context of the complementarity-determining region (CDR) of antibodies, and particularly the hypervariable CDR-H3 loop, a region known for its conformational diversity and critical role in antigen recognition (van Dijk and van de Winkel 2001, Fernández-Quintero *et al.* 2019b). While some successful applications of protein docking have been reported for more rigid systems (Gaudreault *et al.* 2023a) or when experimental information is leveraged (Thornburg *et al.* 2013), it has generally shown limited success for more conformationally diverse proteins, which are inherently more difficult to model, such as antibodies.

In response to these limitations, in a previous study we explored integrating traditional docking methods with AlphaFold (Gaudreault *et al.* 2023b). Physics-based docking tools were used to generate a plethora of structural models, which were then evaluated by AlphaFold, similarly to other studies in related fields (Roney and Ovchinnikov 2022, Ghani *et al.* 2022). Reminiscent of rescoring techniques (Pierce and Weng 2007, Vreven *et al.* 2020), models were re-prioritized using the confidence scores of AlphaFold. The combination of physics-based methods with AlphaFold was shown to outperform AlphaFold-Multimer in antibody-antigen docking scenarios while being less demanding in resources, highlighting the benefits of template-based approaches over template-free modeling approaches. Moreover, we underlined the transformative capability of AlphaFold in remodeling the backbone during the rescoring process, which often improved the quality of the model. This feature not only addressed some of the limitations associated with backbone rigidity but also introduced a valuable compensatory mechanism mitigating the search-scoring trade-off in traditional rigid-docking methods. This advancement marked a significant stride towards overcoming the constraints of rigid docking protocols, and thus it holds promise for more realistic and biologically relevant predictions in the intricate atomic-level landscape of molecular interactions.

Recent years have witnessed a growth in the number of deep-learning-guided antibody modeling tools that can predict antibody structures in their free or unbound states (Abanades *et al.* 2023, Lee *et al.* 2023, Ruffolo *et al.* 2023). While it is a matter of debate whether models of unbound antibodies are valid for antigen docking, there is increased evidence that bound conformations of antibodies are sampled in their unbound states (Fernández-Quintero *et al.* 2019a, 2020, Liu *et al.* 2024). The performance of antibody modeling tools in reproducing binding-competent states on untrained data has significantly improved over the years (Abanades *et al.* 2023). The more traditional antibody modeling tools relied on energy-based scoring functions (Sircar *et al.* 2009, Roy *et al.* 2010) that often failed to discriminate correct models from incorrect ones. One interesting feature of the newer artificial intelligence (AI)-guided modeling tools is their ability to provide models with confidence estimates of the expected error to the ground truth. However, the trustworthiness of confidence estimates has not been clearly demonstrated for traditional physics-based docking models, which generally have been more sensitive to fluctuations in protein backbone conformation.

In a previous performance assessment of AI-enhanced physics-based docking tools, we relied on antibody-antigen systems in which both the bound and the unbound antibody structures were available from X-ray crystallographic studies. This greatly reduced the number of systems available for benchmarking and impacted the statistical significance of that study. Moreover, the full potential of AI-enhanced physics-based tools may not have been exploited by including a single, experimentally determined, conformational state of the antibody CDR and thereby imposing rigid-backbone constraints during docking. Generating multiple structural models of a given antibody for antigen docking would better reflect many real-life applications which start from the antibody amino-acid sequence. To this end, the current study explored AI-generated ensembles of antibody models for use in antigen docking. We benchmarked performance against the latest cutting-edge tools available in this fast-evolving field (Abramson *et al.* 2024, Wohlwend *et al.* 2024). As the antibody-antigen docking methods continue to improve, we emphasize the importance of success metrics in model evaluation and selection for subsequent antibody optimization.

## 2 Materials and methods

### 2.1 Antibody-antigen docking dataset creation

We collected antibody-antigen complexes from SAbDab (Dunbar *et al.* 2014). Only entries that were released from January 1^st^, 2023 and onwards were retained to avoid any overlap with the data that was used to train the software used in this study. A resolution better or equal to 3.0 Å in the crystal structure was imposed. A total of 81 complexes met our criteria and were used as part of the benchmarking. The complete sequences were made available as part of the Supplementary Information.

The antibody models were produced by IgFold (Ruffolo *et al.* 2023), ABodyBuilder2 (Abanades *et al.* 2023), and EquiFold (Lee *et al.* 2023). The tools were run to obtain four antibody models per software. For ABodyBuilder2 and IgFold, one antibody model was predicted for each of the four independently trained deep-learning models. For EquiFold, a single deep-learning model is available. The target of four antibody models was reached by re-running the tool multiple times producing a distinct conformation at every iteration. The antibody models were then energy-minimized with the already built-in minimization protocols within each tool, i.e. OpenMM (Eastman *et al.* 2024) for ABodyBuilder2 and PyRosetta (Chaudhury *et al.* 2010) for IgFold. For models generated by EquiFold, the structures were minimized with an in-house minimization protocol that uses Amber (Cornell *et al.* 1995). Despite some methodological differences, it is not anticipated that minor atomic fluctuations from using different force-fields would impact docking performance.

The antibody models were assigned a certainty value by applying *Z*-scoring to the confidence values of the models, outputted in the form of predicted errors or B-factors by the software. To achieve this, we calculated the arithmetic mean and standard deviation of the errors for all models produced across all antibody-antigen systems. We standardized the errors by subtracting the mean from every predicted error and divided the result by the standard deviation. Because smaller predictor errors are associated with higher confidence, we flipped the sign to obtain the model certainty. The standardization was performed separately for ABodyBuilder2 and IgFold models. Unlike other tools that provide predicted errors, EquiFold does not provide such metrics, therefore, not allowing its models to be assigned a certainty value.

The antigen models were first obtained by rigidly dissociating the antigen from the antibody-antigen crystal structure. To ensure deviation from the bound state, perturbations in the spatial arrangements of side-chains were introduced using SCWRL4 (Krivov *et al.* 2009). This step aimed to simulate a more realistic unbound state of a rigid antigen while reducing the complexity of the analysis and allowing us to focus on how good the antibody structure prediction must be to yield a successful prediction.

### 2.2 Template-based modeling using docking

The complexed states of the antibody-antigen were generated using protein–protein molecular docking software. ProPOSE (Hogues *et al.* 2018) version 1.0.3 and ZDOCK (Chen *et al.* 2003) version 3.0.2 were run to generate the top-100 ranked models per software. The molecules were prepared as previously described (Gaudreault *et al.* 2023b). The docking protocols were run with standard settings while constraining binding to the CDR region. No epitope information was provided.

The antibody-antigen models produced in docking were rescored with AlphaFold version 2.3.1 using the model_1_ptm model with default parameters as previously described (Gaudreault *et al.* 2023b). The docking-generated models were re-ranked according the AF2_ExpComposite_ in Equation (1).

(1) $${\text{AF}2}_{\text{ExpComposite}}=Z_{\text{pLDDT}}+Z_{\text{pTM}}+Z_{\text{ipTM}}+Z_{\text{phys}}.$$

In comparison to the previous rescoring scheme (Gaudreault *et al.* 2023b), the scheme in this study was expanded to include the *Z*_ipTM_ and *Z*_phys_, which are the standardized ipTM and physics-based docking scores, respectively. As AlphaFold does not explicitly output the ipTM score when non-multimeric models are used, the value was manually calculated from the aligned confidence probabilities. All terms were weighted equally in the new scheme to avoid any potential overfit and allow for an increased transferability.

### 2.3 Template-free modeling using AlphaFold and boltz-1

AlphaFold-Multimer (Evans *et al.* 2022) was run through ColabFold version 1.5.2 (Mirdita *et al.* 2022) using the latest multimer_v3 models. For clarity, AlphaFold-Multimer is referred as AlphaFold2 in this paper. Boltz-1 version 0.4.1 was run using the unpaired MSA generated by the ColabFold runs. The AlphaFold2 and Boltz-1 calculations were run on Nvidia A100 Ampere, P100 Pascal and V100 Volta cards on the Digital Research Alliance of Canada clusters. AlphaFold version 3 (AlphaFold3) (Abramson *et al.* 2024) was run on the web server made accessible by its authors. The AI-based models for complex prediction, including AlphaFold-Multimer, AlphaFold3 and Boltz-1, were run five times to generate 5 models per seed, or 25 models in total. Increasing the number of seeds would likely enhance sampling and performance for AI-based models as previously reported (Wallner 2023, Abramson *et al.* 2024), although at a higher computational cost. In contrast, 100 models were used for docking-based tools, which is still considerably lower than the number of models typically evaluated in docking (Huang 2015, Hogues *et al.* 2018). Similarly, increasing the number of docking models to be rescored would likely also improve performance.

### 2.4 Interface properties calculations

Starting from the crystal structures of the antibody-antigen complexes, surface areas (SA) of the residues were calculated. These SAs were then compared to the fully exposed residues to determine the relative surface areas (rSA). The rSA was calculated for each residue in both the complexed (rSA_c_) and uncomplexed (rSA_u_) states, with the latter obtained by a rigid separation of the two molecules. The differences in rSA between these states were computed (ΔrSA). Residues were classified based on their rSA values as follows: interior (rSA_c_ < 25% and ΔrSA = 0%), surface (rSA_c_ > 25% and ΔrSA = 0%), support (rSA_c_ < 25% and ΔrSA > 0%), rim (rSA_c_ > 25% and ΔrSA > 0%), and core (rSA_u_ > 25%, rSA_c_ < 25%, and ΔrSA > 0%). The support, rim, and core regions were defined as the union of the residues in each respective class. Levy scores were calculated by summing the stickiness scores (Levy *et al.* 2012) for all residues within these regions.

The global density (GD) serves as a proxy for determining the atom packing at the interface, specifically for atoms with a ΔrSA greater than 0%. To calculate GD, we first determine the volume of an ellipsoid based on the three moments of inertia from the subset of interface atoms. Then, the number of atoms is divided by the area of an ellipse defined by the two largest moments. The surface complementarity (SC) index is calculated by projecting normal vectors onto the tessellated surface of the interface. Normal vectors that are misaligned are penalized, and the resulting values are weighted by the tessellated area.

## 3 Results

### 3.1 Conformational diversity

**Figure 1.**
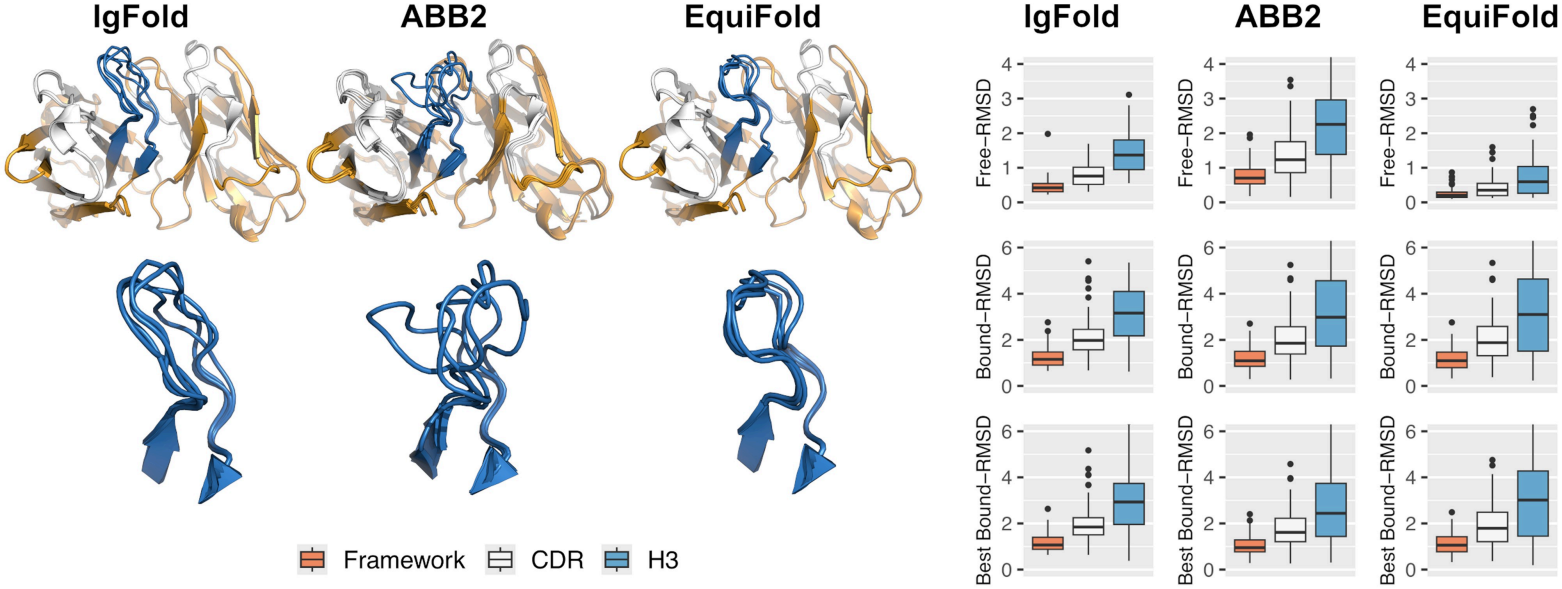
Structural diversity in the predicted antibody ensembles produced by IgFold, ABodyBuilder2, and EquiFold. The structural views depict representative deviations observed within the dataset for the antibody modeling tools. The free-RMSD, derived by pairwise comparisons among all free antibody models present in a given ensemble and averaged over all antibody-antigen systems, highlights the degrees of diversity characterizing the unbound antibody conformational ensembles generated by specific antibody modeling tools. The bound-RMSD describes the deviations of free antibody models to the corresponding antigen-bound antibody crystal structures for all free-antibody models in each ensemble (bound-RMSD) or only for the best free-antibody models which are conformationally closest to the antigen-bound antibody crystal structures (best bound-RMSD). The RMSD values were calculated upon best-fit superposition of the framework region on the subset of backbone atoms, with free-RMSD based on pairwise RMSD between all antibody structures in the model ensembles, and bound-RMSD and best bound-RMSD based on the antigen-bound antibody crystal structures taken as references.

Structural ensembles of free antibodies, i.e. without their antigens, were generated from sequence only for the 81 antibody-antigen systems in the test dataset. To generate conformational diversity in the CDR region of free antibodies, ensembles were modeled using a combination of three state-of-the-art AI-based antibody modeling tools (Fig. 1). Four models were produced with each tool. For each system, we calculated the root-mean-square deviation (RMSD) values between models generated with each tool, called free-RMSD. The extent of CDR conformational diversity varied depending on the tool used. ABodyBuilder2 generated the most heterogeneous ensembles with free-RMSD values of 1.3 and 2.3 Å in the CDR and H3 regions, respectively. These values were accompanied by larger variations also in the framework region. In contrast, the ensembles produced by EquiFold were the most homogeneous, with free-RMSD values of 0.4 and 0.6 Å, respectively. The diversity among IgFold models was intermediate between ABodyBuilder2 and EquiFold.

To assess whether the ensembles could approximate the antibody state in the presence of its antigen, we calculated bound-RMSD values, which are relative to the bound states of antibodies as observed in crystal structures. When including all four models generated by IgFold, ABodyBuilder2, and EquiFold, the bound-RMSD values in the entire CDR were 2.0, 1.9, and 2.0 Å, and the bound-RMSD values in the H3 loop were 3.2, 3.0, and 3.3 Å, respectively. These relatively high values highlight the difficulty of modeling the bound states of antibodies from sequence alone. More noticeable differences between various tools emerged when evaluating the best models produced. ABodyBuilder2 generated models that were closest to the bound states, with overall bound-RMSD of 2.5 Å for the H3 loop compared to 2.9 Å for IgFold and 3.0 Å for EquiFold. The distribution for IgFold is noticeably tighter, signifying that there is less abundance of models closer to the bound state in comparison to EquiFold. These results suggest that ABodyBuilder2-generated ensembles might be better suited for physics-based docking studies due to their superior geometric complementarity to the antigen.

The antigens were initially modeled using the known crystal structure of the antigen in its bound state, with random perturbations introduced to the side-chains. To explore how the models would differ if the antigen structures were unknown, we utilized AlphaFold to generate antigen models and then assessed the degree of conformational change to their bound states (Supplementary Fig. S1). On average, the median RMSD for the entire backbone was 1.3 Å for AlphaFold2 and 1.0 Å for AlphaFold3. Notably, AlphaFold2 and AlphaFold3 performed even better in modeling the interface backbone, achieving RMSDs of 1.0 and 0.7 Å. In terms of side-chain movements, AlphaFold3 models exhibited RMSDs of 1.9 Å, comparable to the 1.6 Å observed in models from SCWRL. However, AlphaFold2 demonstrated less accuracy in modeling interface side-chains, with RMSDs of 2.4 Å, making its structural models less appropriate for the purpose of the present docking study. Therefore, we chose to perform docking against antigens with known bound backbone structure, since introducing large errors in antigen structure may confound the impact of antibody model quality to docking performance, a principal thrust of this study.

### 3.2 Naïve-selection performance

**Figure 2.**
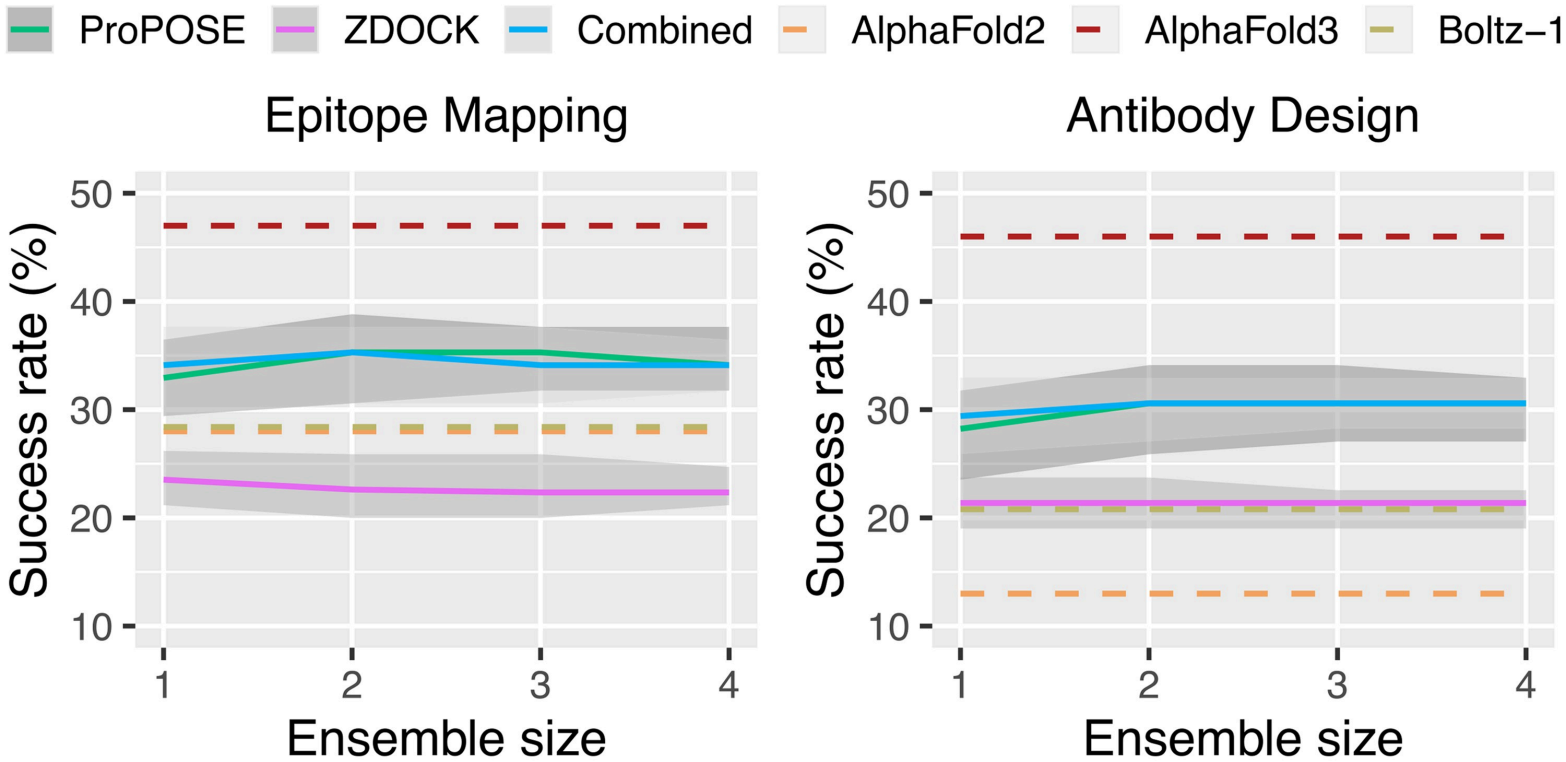
Success rates obtained from a naïve (i.e. random) selection of models for epitope mapping (DockQ≥ 0.23) and antibody design (DockQ≥ 0.49). The rates were plotted as a function of the ensemble size to assess the impact of including an increasingly larger number of free-antibody conformational models. The performances of AI-augmented physics-based tools before (ProPOSE and ZDOCK) and after pooling their results (Combined) are compared to those of AlphaFold-Multimer (AlphaFold2), AlphaFold3, and Boltz-1. The error bars were obtained from bootstrapping the antibody models with replicates for 200 iterations. The top-5 docking predictions using the EquiFold-generated models are shown.

Using physics-based docking tools, we docked the antibody models against their respective antigens and rescored the top-100 predicted poses with AlphaFold2 as previously described (Gaudreault *et al.* 2023b). Two different docking tools were used to explore potential benefits and synergistic effects of combining their predictions. Overall, 194 400 antibody-antigen models were produced, equating to 32 400 per free-antibody conformational ensemble per docking tool. We present the top-5 results based on two applications of docking: (i) for epitope mapping, where a general location of the binding site is sufficient, or (ii) for antibody design, where more precise atomic resolution is required, especially at the antibody-antigen interface, to allow for further development such as affinity maturation. The two applications are associated with specific requirements for model quality. For antibody design, we imposed a requirement for at least a medium-quality model, defined as having a DockQ score of 0.49 or more (Basu and Wallner 2016). In contrast, for epitope mapping, we loosened the criteria and tolerated acceptable-quality models, defined as having a DockQ score of 0.23 or more. The results detailing the prediction quality for each individual antibody-antigen system are provided as part of the Supplementary Information (Supplementary Fig. S2). First, we present docking results when no prior knowledge is available. In this instance, the antibody models are selected naively by randomly choosing a single or multiple structural models. In this naive context, the maximum success rates for AI-enhanced template-based docking were achieved using the EquiFold ensembles with a mean DockQ of 0.19. We noticed minimal to no impact from using a single or multiple models for the free-antibody ensembles. Increasing further the ensemble size up to 10 models did not improve success rates (Supplementary Fig. S3A). The template-based docking tools were able to achieve success rates of up to 35% for epitope mapping consistently outperforming the template-free docking tools, AlphaFold2 and Boltz-1, that achieve 28%, but unable to match the 47% of AlphaFold3 (Fig. 2). When moving to the antibody design scenario, success rates are maintained for AlphaFold3 at 46%, the template-based docking tools drops to 30%, with lower success rates for AlphaFold2 at 13%, and Boltz-1 at 21%. It is clear that Boltz-1 and AlphaFold2 share similarities in their successful systems (Supplementary Fig. S2). On average, Boltz-1 produced higher quality models (mean DockQ of 0.23) when compared to AlphaFold2 (mean DockQ of 0.20) closely matching results from an independent study (Boitreaud *et al.* 2024).

In both applications, the performance of AlphaFold3 exceeded the one of AI-augmented physics-based docking predictions, underscoring the substantial improvements in interface modeling from AlphaFold2 to AlphaFold3. When restricting the analyses to a single replicate, AlphaFold3 achieves equivalent numbers to a separate benchmark study (Hitawala and Gray 2025) with 42% and 35%.

When using the IgFold and ABodyBuilder2 ensembles, performances were consistently lower and the impact on the number of models was more pronounced (Supplementary Fig. S3B and C). Interestingly, although ABodyBuilder2 produces the best antibody models on average (Fig. 1), its ensembles are the most heterogeneous in CDR conformational diversity and impact performance negatively. In line with these results, pooling the different ensembles into a single one also impacted performance negatively (Supplementary Fig. S3D), implying a need for the prioritization of models.

### 3.3 Confidence-guided performance

**Figure 3.**
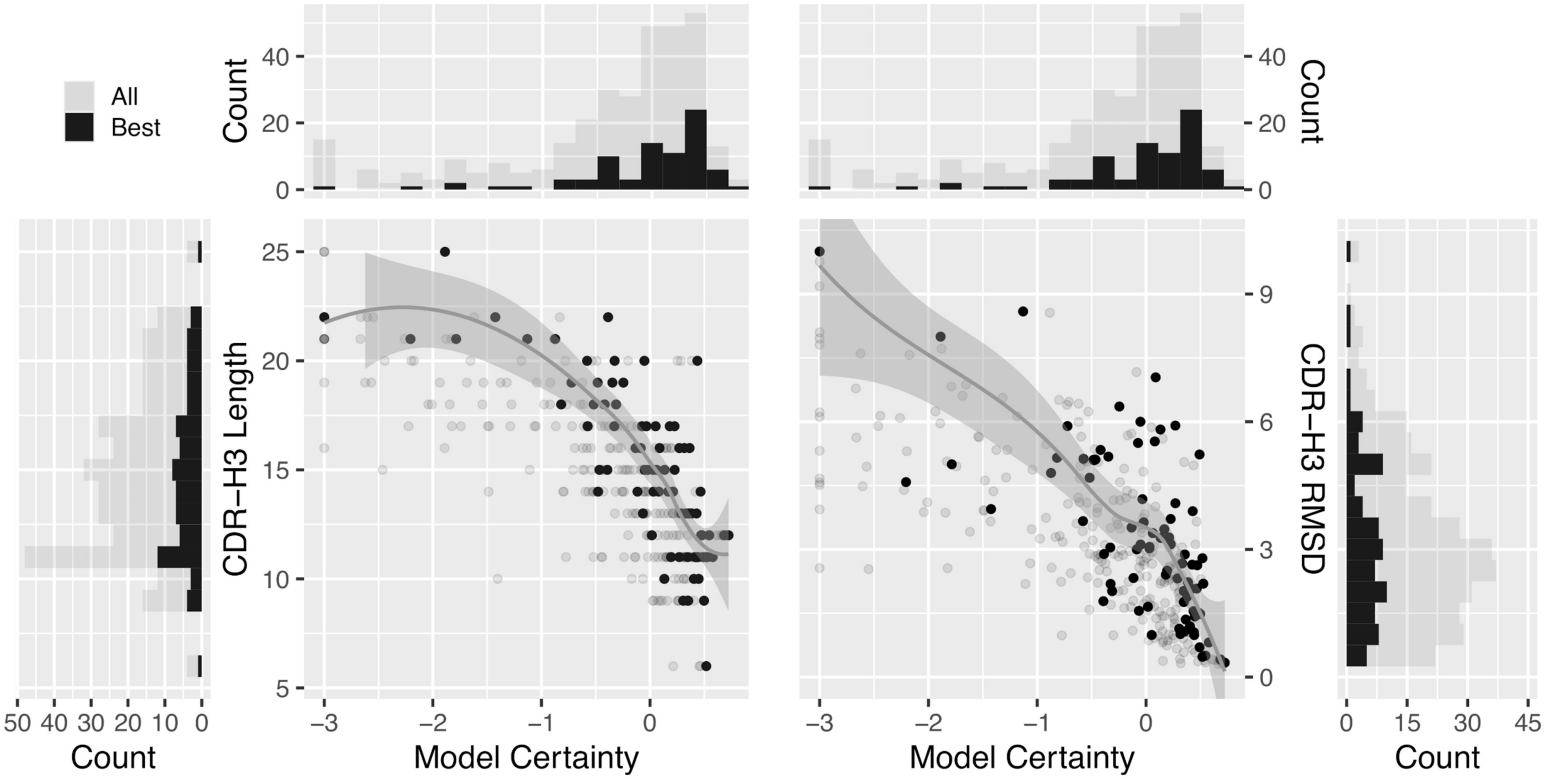
Standardized confidence scores plotted as model certainty against the length in CDR-H3 and RMS deviations to the CDR-H3 in the bound state, for the ABodyBuilder2 ensembles. The smoothed regression lines were built from the best subset of models, i.e. only considering the model with highest certainty for the antibody-antigen systems. The Pearson correlations (*R*^2^) for the best models are 0.58 and 0.47 for the CDR-H3 length and RMSD, respectively. Histograms were plotted to aid in visualizing the density of points in model certainty (top), CDR-H3 length (left), and CDR-H3 RMSD (right). Each bar corresponds to 0.2 certainty units, to 1 residue, or to 0.5 Å, respectively.

Although the naive model selection approach is informative, it is not the most practical approach. Historically, physics-based functions were used to prioritize and select models based on energies, but these often failed to effectively discriminate between good and poor models. Instead, AI-guided modeling tools now provide us with confidence values for their predicted models, indicating how closely the models are projected to match the reference structures. We investigated the accuracy and reliability of the confidence values and explored how they could be leveraged for improved docking predictions. The analysis excludes EquiFold ensembles due to the lack of confidence values for its models. To facilitate the comparison between confidence scores among ensembles, we standardized the confidence scores on a scale of model certainty (Fig. 3). We noticed a correlation between the length of the CDR-H3 loop with model certainty from the ABodyBuilder2 ensembles. While there is no guarantee that short CDR-H3 loops will be predicted with high confidence, the likelihood is relatively strong with a Pearson *R*^2^ of 0.58. Similar data were observed on the IgFold ensembles (Supplementary Fig. S4), but with weaker correlation (*R*^2^ = 0.36). A strong correlation was also found between CDR-H3 RMSD to the bound state, a proxy for model quality, with model certainty (*R*^2^ = 0.47) using the ABodyBuilder2 generated models. This suggests that model certainty could be used to prioritize models by filtering out poor-quality models. This may in turn reduce noise from the inclusion of too many models and thereby improve performance.

**Figure 4.**
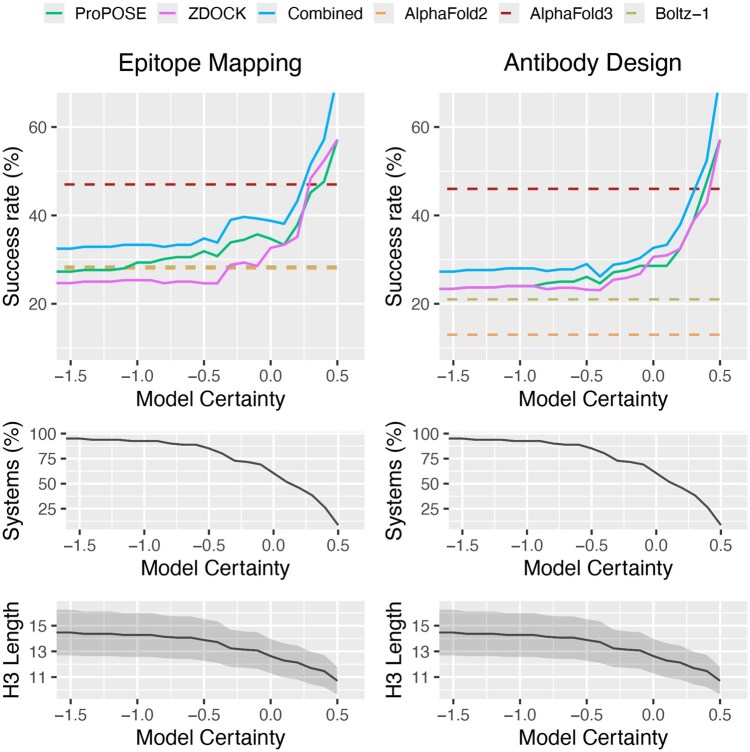
Success rates obtained from a confidence-guided selection of models for epitope mapping (DockQ ≥ 0.23) and antibody design (DockQ ≥ 0.49). The rates were plotted as a function of the model certainty threshold below which antibody models are rejected. The performance of AI-augmented physics-based tools before (ProPOSE and ZDOCK) and after pooling their results (Combined) is compared to the ones of AlphaFold-Multimer (AlphaFold2), AlphaFold3, and Boltz-1. For transparency, the number of systems remaining with their average length in CDR-H3 are reported. A minimum representation of 5% was imposed for data points to be plotted to minimize abruptness from the impact of low sample size. The top-5 docking predictions using the ABodyBuilder2-generated models are shown when using AbodyBuilder2-generated models.

We tested this hypothesis and calculated the success rates while thresholding the input model quality to reject uncertain models predicted to be of lower quality. The performance in epitope mapping and antibody design are shown for the ABodyBuilder2 ensembles (Fig. 4). With low stringency, success rates using the AI-augmented physics-based approach 32% in epitope mapping, just slightly better than AlphaFold2 and Boltz-1 but still inferior to the naive selection from the EquiFold-generated models. We observed significant improvements in success rates as we increased the stringency of the threshold. While improvements in success were also noticeable using the IgFold ensembles, performance was generally lower (Supplementary Fig. S5A). Thresholding comes at a cost of reduced sample size: as the threshold becomes more stringent, more systems are excluded. In both epitope mapping and antibody design, the success rates reach those of AlphaFold3 when ∼50% of systems are retained (*N* = 42), coinciding with an average CDR-H3 length of 12. At 25% systems remaining (*N* = 21), the performance of AlphaFold2-enhanced physics-based tools achieves 53% and 50% success in epitope mapping and antibody design, respectively. While it would be ideal for docking to be successful across the full range of CDR lengths, this is currently not feasible even with state-of-the-art antibody modeling tools. Nonetheless, our results highlight a specific applicability range for docking tools, providing valuable insights for future improvements.

While it may appear unfair to calculate success rates on different subsets of the dataset and compare those obtained by AlphaFold on the complete dataset, our approach mirrors how we would typically use AlphaFold, i.e. modeling from sequence alone without applying stringency based on an initial structural model quality. If we were to apply thresholding to AlphaFold in a similar manner, we would find that AlphaFold performance is not impacted on the different subsets. Provided the high correlation of model certainty with CDR-H3 length, this suggests that AlphaFold is less sensitive to CDR-H3 length than physics-based tools (Supplementary Fig. S5B) and that applying such a thresholding strategy would not enhance the performance for AlphaFold.

### 3.4 Discriminants for success

**Figure 5.**
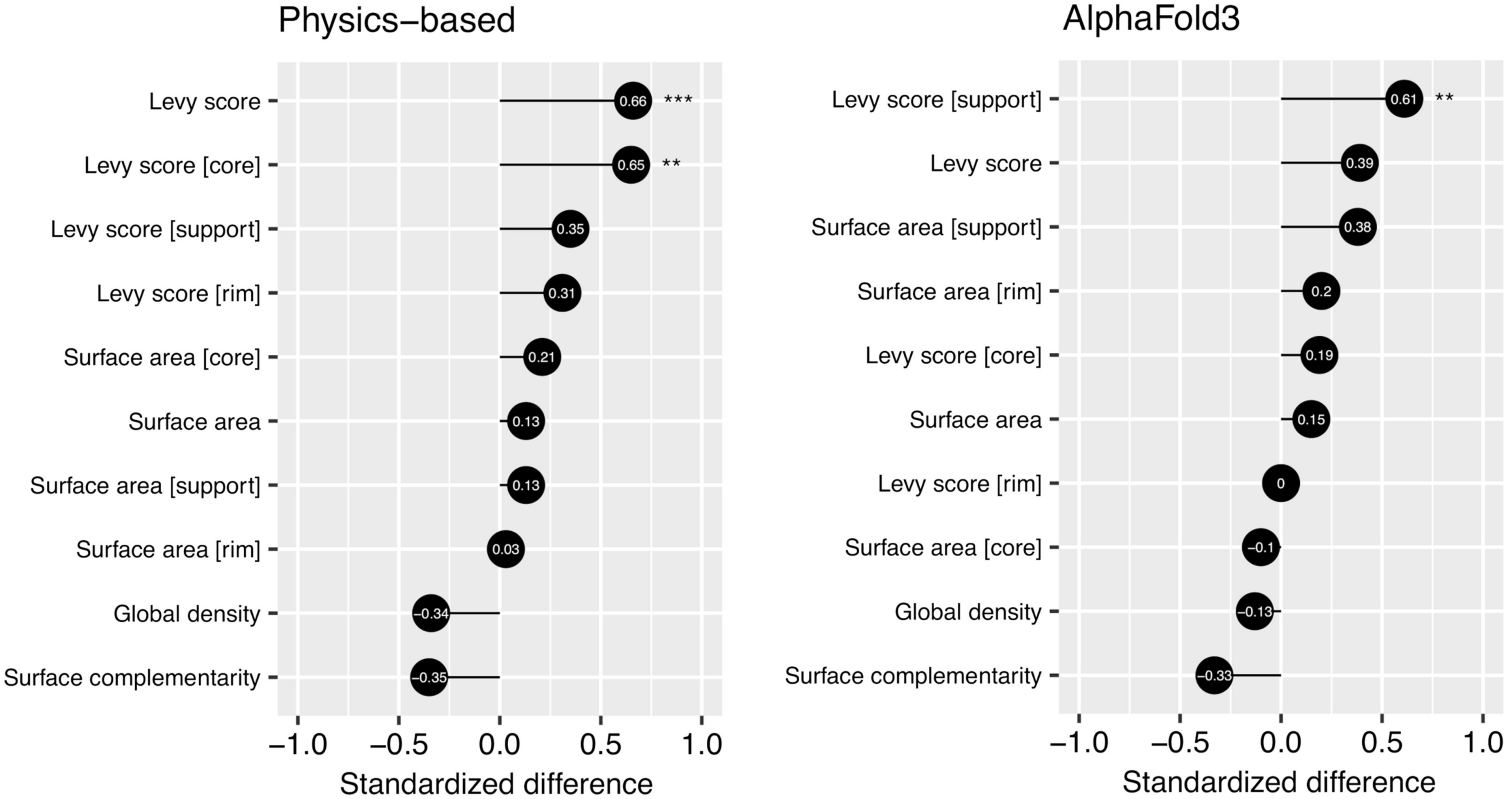
Standardized differences for a panel of properties characterizing antibody-antigen interfaces of crystal structures. The properties were calculated for models generated by physics-based tools and AlphaFold3 in the antibody design regime. The differences were calculated by comparing the mean of the subset of complexes that could be successfully predicted within the top-5 predictions to the mean of the subset of failures. Successes for physics-based tools are defined as the union of the successes across all tools used. *P*-values were calculated using t-tests from the underlying distributions of successes and failures. The significance of the *P*-values is indicated as follows: *P* < .05 (*); *P* < .01 (**), and *P* < .001 (***). Positive standardized difference values indicate higher success when the property is high and negative differences indicate higher success when the property is low.

In an effort to find indicators for successful prediction, we characterized the antibody-antigen interfaces. We examined the whole interface as well as the support, core and rim regions that define the interface, according to previously described definitions (Levy 2010). These regions were analyzed in terms of size, through SA calculations, and in terms of chemistry, through the stickiness score from Levy *et al.* (2012). Sticky interfaces are commonly observed in protein–protein interfaces due to their composition in amino acids, which is primarily characterized by the presence of hydrophobic groups. We complemented the analysis with the GD and surface complementarity index, properties that describe the packing of atoms at the interface, and the complementarity in molecular surface between the antibody and the antigen (Prasad Bahadur *et al.* 2004). The properties were compared between successes and failures of the physics-based tools (Fig. 5). It is clear that the amino acids that compose the binding interface is a strong determinant for success. Physics-based tools tend to fail more often for interfaces that are less sticky (*P* < .001), primarily from the core region of the interface (*P* < .01). Previous studies have raised the importance of SA as a major contributor for success in rigid protein–protein docking, with smaller interfaces being more challenging to predict (Hogues *et al.* 2018). This trend becomes increasingly more evident as we increase stringency on model certainty, retaining only those models that approach their bound states and thereby mimicking closely the rigid docking experiment (Supplementary Fig. S6); the SA properties all emerge as significant. The properties were also analyzed for successes and failures of AlphaFold3 (Fig. 5). AlphaFold3 fails more often on systems with support regions that are less sticky (*P* < .01). The GD and surface complementarity index did not come up as significant determinants in the analyses.

## 4 Discussion

In this study, we benchmarked AI-augmented physics-based docking tools alongside novel AI modeling technologies including AlphaFold and Boltz-1. Certainly, a major challenge of the study was to objectively compare tools that operate with fundamentally different workflows. In the case of deep-learning utilities, the tools start from sequence data, whereas traditional docking tools start from known structures. This difference alone can introduce some bias due to existing experimental structural knowledge in one case. The quality of the input structures is undeniably an important factor to the success of docking tools. Precise atomic accuracy of both antigen and antibody models is essential considering that docking relies on good geometric and physicochemical complementarity. As expected, the success of docking is influenced by the quality of the input antibody models, with a noticeable enrichment in success when models are closer to bound structures (Supplementary Fig. S7). ABodyBuilders2 produced the best models overall, resulting in a higher number of high-quality complexes (Supplementary Fig. S2). However, its overall success was still lower than the one of EquiFold, suggesting an impact on the nature of the ensemble. EquiFold showed little to no impact on success even when more models were introduced that display limited diversity in CDR conformational variability. In contrast, the heterogeneous ensembles from ABodyBuilder2 included models significantly different from bound states, affecting success when used in docking. Proximity to bound states did not always ensure success, as various factors, such as a single obstructive side-chain, can prevent docking from generating high-quality models that would be preferred for AlphaFold2 rescoring to achieve high confidence scores akin to those from crystal structures (Gaudreault *et al.* 2023b). While some models were effective enough to succeed, AlphaFold2 rescoring confidence scores were diluted by less accurate models. This highlights the need for antibody model prioritization, especially when CDR variability is high, despite the drawbacks of thresholding that can lead to the rejection of certain systems. Perhaps the use of AlphaFold3 as a rescoring utility instead would provide more useful results and be less subject to noise, but this remains to be investigated and involves a whole development work to evaluate the feasibility.

In discovery campaigns, often the structure of the antigen is already known, making it relevant to test tools under such conditions. One bias of our study, however, might have been that we chose to report docking performance using antigen structures with known bound backbone conformations, which likely impacted favorably the docking results, but allowed us to tease out the importance of the antibody structure prediction when used for docking. Perhaps a more difficult but objective evaluation of docking might have been one starting from models generated with AlphaFold, as these models also introduce deviations in the backbone structure. Our indirect evidence suggests that docking performance using AlphaFold3-generated models would not be as drastically impacted as anticipated; AlphaFold3 produced models with sub-1 Å average deviations in the interface backbone atoms that show comparable side-chain movements than the antigen models used in the benchmark. It is fair to say that some performance would have been lost if AlphaFold3 models had been used instead. If that would be the case, the performances of our AI-enhanced physics-based approaches in epitope mapping may approach those of AlphaFold2 and Boltz-1. In antibody design, however, given the larger differences in success, especially for AlphaFold2, the use of docking-based approaches should still be preferred. It is undeniable that the utility range of physics-based docking approaches has narrowed with the emergence of AlphaFold3 that achieves unmatched performance (Abramson *et al.* 2024). We believe physics-based docking should only be used in cases where a confident model of the antibody can be produced, otherwise one should resort to the more accurate AI-technologies for prediction of protein complexes. While Boltz-1 has similar infrastructure and provides current advantages over AlphaFold3 such as open-access code with unrestricted use, its reported performance is still much lower when compared to AlphaFold3 for antibody-antigen complex prediction.

Benchmarking presents complexities, particularly in controlling the parameter space, as numerous parameters can impact results. Exploring the entire parameter space is often limited by practical constraints, typically relying on default values as users normally would. It would be impractical to optimize parameters solely to fit a benchmark dataset. While MSA resampling was shown that it can improve predictability in some applications (Abramson *et al.* 2024, Wayment-Steele *et al.* 2024), it greatly limits the throughout from the much higher computational cost. Another critical parameter is the availability of experimental information, which could narrow down the number of epitopes to evaluate and improve success (Boitreaud *et al.* 2024). In this study, we focused on antibodies binding through their CDR regions without relying on experimental knowledge for the epitope. These considerations highlight the intricacies of benchmarking and underscore the importance of context-specific evaluations.

## Supplementary Material

btaf129_Supplementary_Data

## Data Availability

The AF2 rescoring scripts are available at github.com/gaudreaultfnrc/AF2-Rescoring.
