# Supplementary material for: AI-augmented physics-based docking for antibody-antigen complex prediction
Source: Bioinformatics. 2025 Mar 26;41(4):btaf129. doi: 10.1093/bioinformatics/btaf129 (PMC11978387; doi:10.1093/bioinformatics/btaf129)
Supplement: btaf129_Supplementary_Data [file btaf129_supplementary_data.zip › AF2-AbModels_Manuscript_SI_Bioinformatics_Corrected_Final.docx]

*Supplementary Information*

**AI-Augmented Physics-Based Docking
for Antibody-Antigen Complex Prediction**

**Francis Gaudreault^1^, Traian Sulea^1,2^ and Christopher R. Corbeil^1,3*^**

^1^ Human Health Therapeutics Research Centre, National Research Council Canada, 6100 Royalmount, Avenue, Montreal, Quebec, Canada, H4P 2R2

^2^ Institute of Parasitology, McGill University, 21111 Lakeshore Road, Sainte‑Anne‑de‑Bellevue, Quebec, Canada, H9X 3V9

^3^ Department of Biochemistry, McGill University, 3649 Promenade Sir-William-Osler, Montreal, Quebec, Canada, H3A 1A3

^*^ Corresponding author: [christopher.corbeil@nrc-cnrc.gc.ca](mailto:traian.sulea@nrc-cnrc.gc.ca)


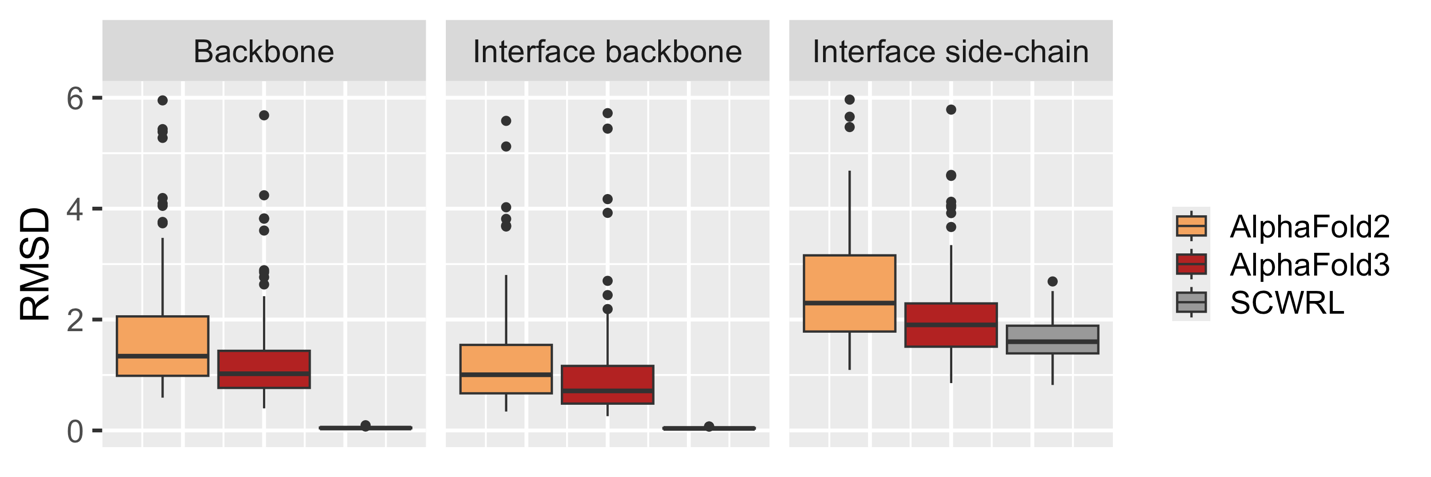


**Figure S1**. RMSD deviations for AlphaFold-Multimer (AlphaFold2), AlphaFold3 and SCWRL-generated models of the antigen alone to the known crystal structure of the bound antigen. The models were modeled starting from sequence data (for AlphaFold2 and AlphaFold3) and from structural data of the bound antigen (for SCWRL). The RMSD values were computed on the subset of backbone atoms following best fit superposition of the whole structure, as well as on the subset of interface backbone and side-chain atoms following best fit superposition of the interface atoms. Interface atoms were defined as those atoms contained within a sphere of radius 5Å from the antibody.

**
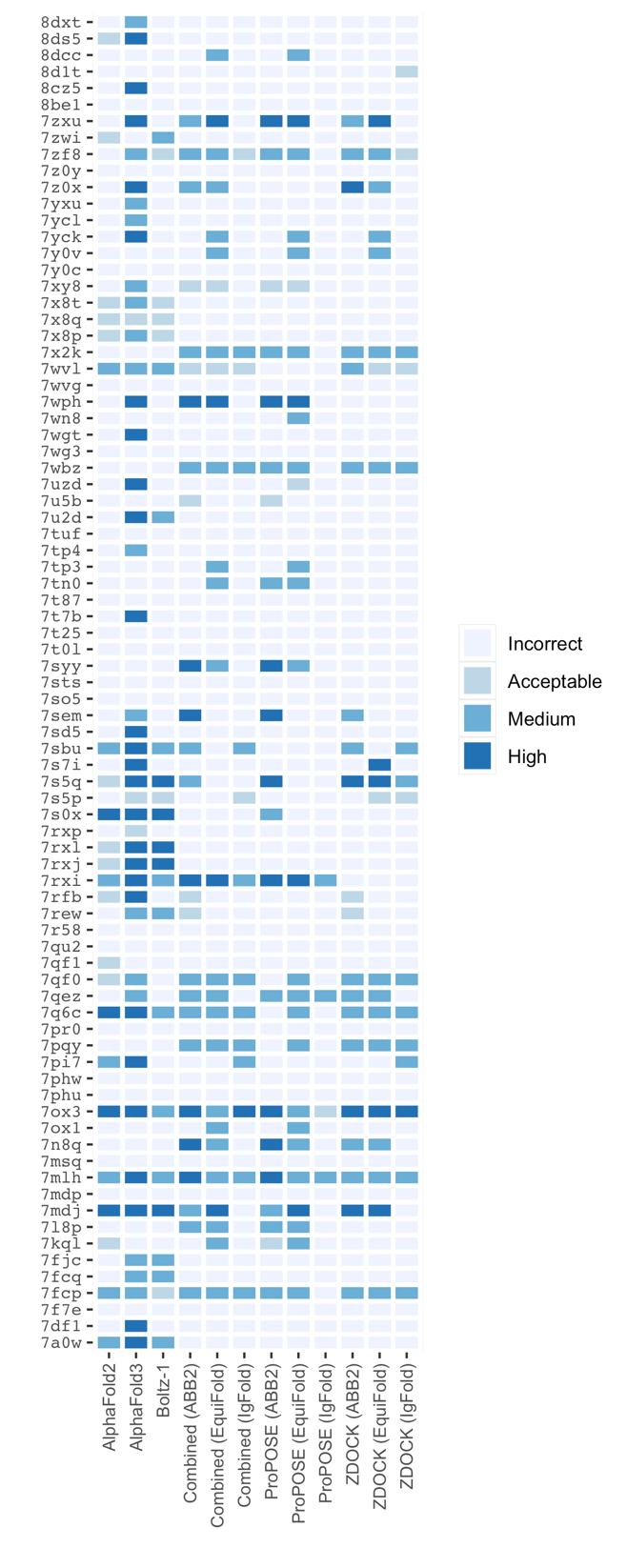
**

**Figure S2**. Results obtained for the various modeling protocols using AlphaFold-Multimer (AlphaFold2), AlphaFold3, Boltz-1 and AlphaFold2-rescored physics-based docking approaches. The results are shown before (ProPOSE and ZDOCK) and after pooling (Combined) using the ABobyBuilder2 (ABB2), IgFold and EquiFold antibody models. The DockQ score thresholds used were 0.23 (Acceptable), 0.49 (Medium) and 0.80 (High).

**A**

**B**

**C**

**D**

**Figure S3**. Success rates obtained from a naive selection of models for epitope mapping (DockQ$\geq$0.23) and antibody design (DockQ$\geq$0.49). The rates were plotted as a function of the ensemble size to assess the impact of including an increasingly larger number of models. The performance of AI-augmented physics-based tools before (ProPOSE and ZDOCK) and after pooling their results (Combined) is compared to the ones of AlphaFold-Multimer (AlphaFold2), AlphaFold3 and Boltz-1. The error bars were obtained from bootstrapping the antibody models with replicates for 200 iterations. The top-5 docking predictions are shown when using (**A**) the expanded EquiFold ensemble, (**B**) the IgFold-generated ensemble, (**C**) the ABodyBuilder2-generated ensemble and (**D**) the combination of the three ensembles.

**Figure S4**. Standardized confidence scores plotted as model certainty against the length in CDR-H3 and RMS deviations to the CDR-H3 in the bound state, for the IgFold ensembles. The smoothed regression lines were built from the best subset of models, i.e. only considering the model with highest certainty for the antibody-antigen systems. The Pearson correlations (R^2^) for the best models are 0.35 and 0.16 for the CDR-H3 length and RMSD, respectively. Histograms were plotted to aid in visualizing the density of points in model certainty (top), CDR-H3 length (left) and CDR-H3 RMSD (right). Each bar corresponds to one unit in 0.2 certainty, 1 residue and 0.5 Å.

**A**

**B**

**Figure S5**. Success rates obtained from a confidence-guided selection of models for epitope mapping (DockQ$\geq$0.23) and antibody design (DockQ$\geq$0.49). The rates were plotted as a function of the model certainty threshold below which antibody models are rejected. The performance of AI-augmented physics-based tools before (ProPOSE and ZDOCK) and after pooling their results (Combined) is compared to the ones of AlphaFold-Multimer (AlphaFold2), AlphaFold3 and Boltz-1. For transparency, the number of systems remaining with their average length in CDR-H3 are reported. A minimum representation of 5% was imposed for data points to be plotted to minimize abruptness from the impact of low sample size. The top-5 docking predictions are shown when using (**A**) the IgFold-generated and (**B**) the ABodyBuilder2-generated models while thresholding to AlphaFold.

**Figure S6**. Standardized differences for a panel of properties characterizing antibody-antigen interfaces from crystal structures. The properties were calculated at 50% and 25% of remaining systems after excluding ABodyBuilder2-generated models with poor confidence. The differences were calculated by comparing the means between the subset of complexes that could be successfully predicted within the top 5 predictions to the subset of failures. Successes are defined as union of the successes across all physics-based tools used. P-values were calculated using t-tests from the underlying distributions of successes and failures. The significance of the p-values is indicated as follows: p < 0.05 (*); p < 0.01 (**) and p < 0.001 (***). Positive standardized difference values indicate higher success when that property is high and negative differences indicate higher success when the property is low.


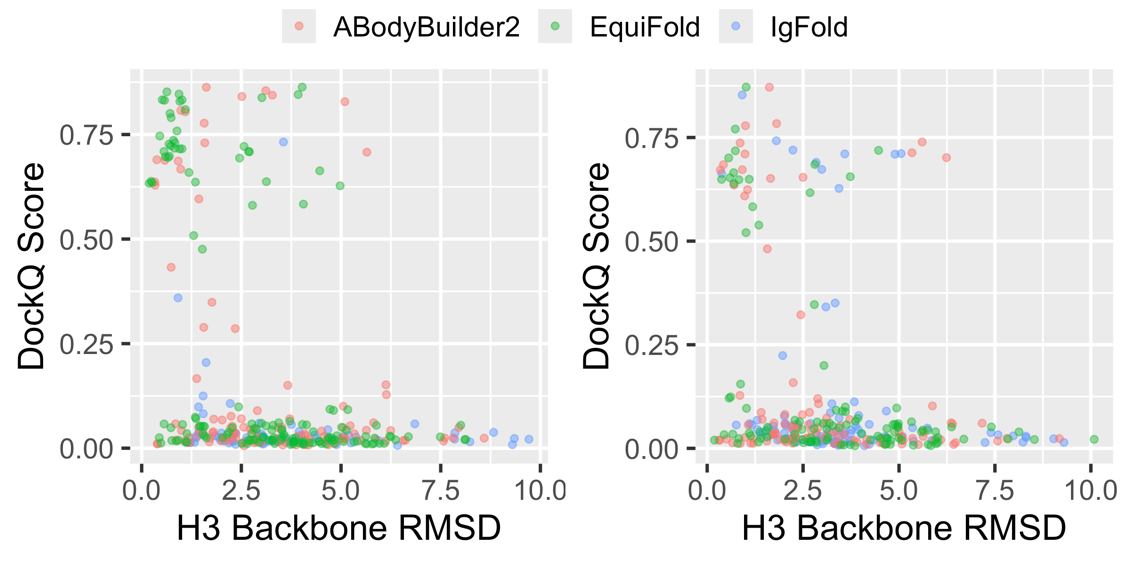


**Figure S7**. Complex prediction quality as a function of backbone deviations in the CDR-H3 loop for all antibody models generated by IgFold, ABodyBuilder2 and EquiFold. The antibody-antigen models were generated employing AI-enhanced physics-based approaches using ProPOSE (left) and ZDOCK (right). The DockQ score is used as proxy for prediction quality.
